# Phospho‐LATS1/2 Immunoreactivity and Survival in Muscle‐Invasive Bladder Cancer: A Quantitative Immunohistochemical Study

**DOI:** 10.1111/iju.70715

**Published:** 2026-11-13

**Authors:** Shota Kakita, Tomohiro Matsuo, Asato Otsubo, Kyohei Araki, Hiroyuki Honda, Shintaro Mori, Kensuke Mitsunari, Kojiro Ohba, Ryoichi Imamura

**Affiliations:** ^1^ Department of Urology, Graduate School of Biomedical Sciences Nagasaki University Nagasaki Japan

**Keywords:** biomarkers, cystectomy, hippo signaling pathway, immunohistochemistry, tumor, urinary bladder neoplasms

## Abstract

**Objectives:**

To evaluate the association between phosphorylated large tumor suppressor kinases 1 and 2 immunoreactivity and survival after radical cystectomy in patients with muscle‐invasive bladder cancer.

**Methods:**

This retrospective study included 96 patients whose pretreatment transurethral resection specimens underwent phospho‐specific immunohistochemistry and digital *H*‐score quantification. Associations with overall survival and metastasis or death after cystectomy were evaluated using multivariable Cox models. Continuous scores and median‐defined low/high groups were analyzed separately. Models incorporating lymphovascular invasion and surgical margin status included 95 patients. Nuclear Yes‐associated protein staining and paired transurethral resection–cystectomy specimens were additionally evaluated.

**Results:**

During a median follow‐up of 67 months, 43 patients died and 47 experienced metastasis or death. Each 10‐point increase in *H*‐score was associated with lower mortality (hazard ratio, 0.956; 95% confidence interval, 0.920–0.993; *p* = 0.020) and a lower risk of metastasis or death (hazard ratio, 0.962; 95% confidence interval, 0.929–0.996; *p* = 0.029). The adjusted low‐versus‐high mortality estimate was imprecise (hazard ratio, 1.777; 95% confidence interval, 0.871–3.624; *p* = 0.114). Nuclear Yes‐associated protein scores were higher in the low‐staining group (*p* = 0.009). Among 71 paired specimens, *H*‐scores were positively correlated (Spearman's *ρ* = 0.520), with 76.1% categorical agreement.

**Conclusions:**

Lower phosphorylated large tumor suppressor kinases 1 and 2 *H*‐scores were associated with shorter overall survival after radical cystectomy. These exploratory findings warrant independent validation, with residual confounding and uncertainty in binary‐group estimates limiting clinical interpretation.

## 1 Introduction

Muscle‐invasive bladder cancer (MIBC) carries substantial recurrence and mortality risks despite radical cystectomy (RC) and perioperative systemic therapy [1, 2]. Pathological stage, nodal status, lymphovascular invasion (LVI), and positive soft‐tissue margins remain important prognostic factors [3, 4]. Clinicopathological nomograms combine clinical and pathological features to estimate postoperative recurrence risk [5], providing a benchmark against which additional markers should be evaluated. Diagnostic transurethral resection (TUR) tissue could provide biological information before systemic treatment, when RC pathology is unavailable. However, an association observed after adjustment for postoperative factors does not establish that the same assay improves pretreatment decisions.

Large tumor suppressor kinases 1 and 2 (LATS1/2) regulate Yes‐associated protein (YAP) and transcriptional coactivator with PDZ‐binding motif (TAZ) within the Hippo pathway [6, 7]. LATS‐mediated phosphorylation restrains YAP/TAZ‐dependent transcription [8]. Experimental bladder‐cancer studies implicate YAP in treatment resistance and progression [9, 10], but clinical immunohistochemical findings are inconsistent: YAP1 overexpression was associated with poor outcomes in one urothelial cohort [11], whereas compartment‐specific YAP1 and phosphorylated YAP1 staining lacked prognostic associations in another [12]. Inhibition of LATS by NUAK family kinase 2 (NUAK2) can sustain YAP/TAZ signaling experimentally, with NUAK2 expression and YAP/TAZ‐associated transcription reported in aggressive bladder tumors [13]. These observations make analyte identity and cellular localization central to interpretation. Phosphorylation of LATS1 at Thr1079 and LATS2 at Thr1041 participates in kinase activation [14, 15]; tissue immunoreactivity at these sites nevertheless differs from total protein abundance and direct enzymatic activity.

In this study, we investigated the association between pretreatment TUR phosphorylated LATS1/2 (p‐LATS1/2) immunoreactivity and survival after RC in patients with MIBC. We evaluated continuous *H*‐scores and median‐defined low/high groups in relation to overall survival (OS) and progression‐free survival (PFS), with adjustment for established pathological prognostic factors. Nuclear YAP staining and paired TUR–RC comparisons were used to explore tissue‐level associations and consistency between specimen types. Together, these analyses aimed to characterize the prognostic relevance of p‐LATS1/2 immunoreactivity and inform its further evaluation as a candidate biomarker.

## 2 Methods

### 2.1 Study Design and Patients

This single‐center retrospective study included 96 patients with clinically or pathologically assessed MIBC undergoing RC between January 2008 and December 2024, with available diagnostic TUR tissue. Clinical staging used bladder magnetic resonance imaging (MRI), computed tomography (CT), and bone scintigraphy. Clinical stage and RC pathological stage (p/yp) were recorded separately. CT/MRI review reclassified 13 initially recorded cT1 cases as cT2a.

Nagasaki University Hospital's Institutional Review Board approved the study (No. 2312815). The study followed the Declaration of Helsinki; written informed consent was waived, and an opt‐out procedure was implemented.

### 2.2 Immunohistochemistry and Image Analysis

Immunohistochemistry used 4‐μm diagnostic TUR sections obtained before systemic therapy. Matched RC specimens with residual tumor were assessed; ypT0 or unavailable RC tissue precluded paired assessment but not survival analysis.

The phospho‐specific antibody (Abcam, ab111344, 1:200) recognizes LATS1 Thr1079 and LATS2 Thr1041 phosphorylation, yielding combined p‐LATS1/2 immunoreactivity rather than total LATS1/2. Nuclear YAP, Ki‐67, CD34, and D2‐40 were assessed. Retrieval, antibody, and detection conditions are detailed in Supplementary Methods.

QuPath 0.5.1 [16] quantified selected tumor fields, including representative invasive fronts. Cytoplasmic p‐LATS1/2 and nuclear YAP intensities were categorized using optical‐density thresholds of 0.20, 0.25, and 0.30. *H*‐scores (0–300) were calculated from tumor‐cell percentages [17, 18]: *H*‐score = (1 × % weak) + (2 × % moderate) + (3 × % strong). The reported median cutoff of 77 and median of approximately 77.352 yielded identical groups of 48 patients each. Restricted analyses retained these assignments. Paired RC scoring used the same protocol; tumor core and invasive front were not separately annotated.

### 2.3 Proliferative and Vascular Indices

Ki‐67 was the percentage of positive nuclei among ≥ 500 tumor cells in a × 400 hotspot. Microvessel density (MVD; CD34) and lymphatic vessel density (LVD; D2‐40) averaged counts from three ×200 hotspots. Stained endothelial clusters were counted regardless of lumen formation; large muscular vessels were excluded. These indices were distinguished from histological LVI.

Two investigators (SK and TM), blinded to clinicopathological information, independently assessed immunohistochemistry and resolved disagreements by consensus. RC‐specimen LVI and surgical margin status were recorded separately from TUR findings.

### 2.4 Statistical Analysis

Nuclear YAP scores were compared between low/high p‐LATS1/2 groups (Mann–Whitney *U* test). Spearman correlation assessed paired TUR–RC p‐LATS1/2 *H*‐scores; categorical agreement assessed classifications at cutoff 77. OS was measured from RC to all‐cause death; PFS was defined as the time from RC to first recorded metastasis or all‐cause death. Local‐recurrence‐only events were not captured in the available dataset. Event‐free patients were censored at last documented progression‐free follow‐up for PFS and last survival follow‐up for OS. Kaplan–Meier, log‐rank, and reverse Kaplan–Meier methods summarized survival and follow‐up.

One metastasis initially recorded at zero months was analyzed at approximately 20 days (0.66 months) after RC.

Cox models used Efron ties and model‐based Wald 95% confidence intervals (CIs). The base model included age, p/ypT3–4 versus 0–2, p/ypN‐positive versus 0, Ki‐67, and continuous p‐LATS1/2. Extended models added RC‐LVI and margins in 95 complete cases; base models used both these same cases and all 96 patients. No imputation was used. Binary models replaced continuous scores with low/high status, with high as reference. Primary‐model continuous p‐LATS1/2 hazard ratios (HRs) were reported per 10 *H*‐score points. Exploratory OS interactions added neoadjuvant chemotherapy (NAC) and p‐LATS1/2‐by‐NAC to the base model.

Proportional‐hazards, restricted‐cubic‐spline, and leave‐one‐out diagnostics assessed assumptions and influence (Supplementary Methods). One RC‐LVI record with discordant composite/component fields retained its positive total‐LVI status in primary models and was excluded in sensitivity analysis. No outcome‐driven covariate selection was used.

Exploratory RC‐landmark propensity‐score matching was specified during revision before inspecting matching results. Logistic scores modeled low‐group membership using age, sex, clinical T3–4, clinical node positivity, and NAC. Matching used 1:1 nearest neighbors, common logit support, no replacement, and a caliper of 0.2 pooled within‐group standard deviations of the logit propensity score [19]. Matched survival inference was not performed if any propensity‐model covariate had an absolute standardized mean difference ≥ 0.10 after matching.

Exploratory 36‐month OS discrimination used inverse‐probability‐of‐censoring‐weighted receiver operating characteristic analysis [20], with lower scores indicating higher risk. Youden‐index cutoff selection required ≥ 20 observations per group. Censoring estimation and selection were repeated in 2000 patient bootstraps to calculate the area under the curve (AUC), its percentile 95% CI, and optimism‐corrected selected‐cutoff performance [21].

### 2.5 Statistical Software and Manuscript Preparation

All final statistical analyses were performed using EZR version 1.68. Figures were prepared using Adobe Illustrator and Matplotlib version 3.10.8. An OpenAI large language model assisted manuscript preparation and analysis‐code generation/checking; authors supplied or approved clinical inputs and analytical choices. Survival *p* values were two‐sided and descriptive, without multiplicity adjustment.

## 3 Results

### 3.1 Patient Characteristics and Immunohistochemical Findings

The cohort included 48 low‐ and 48 high‐staining patients (Figure 1; Table 1). There were 34 versus 45 men and 36 versus 26 NAC recipients, respectively. RC p/ypT3–4 disease was present in 23/48 versus 8/48 patients. RC‐LVI and positive margins were present in 36/95 and 10/95 evaluable patients, respectively. Baseline and RC variant histology were reported separately.

**FIGURE 1:**
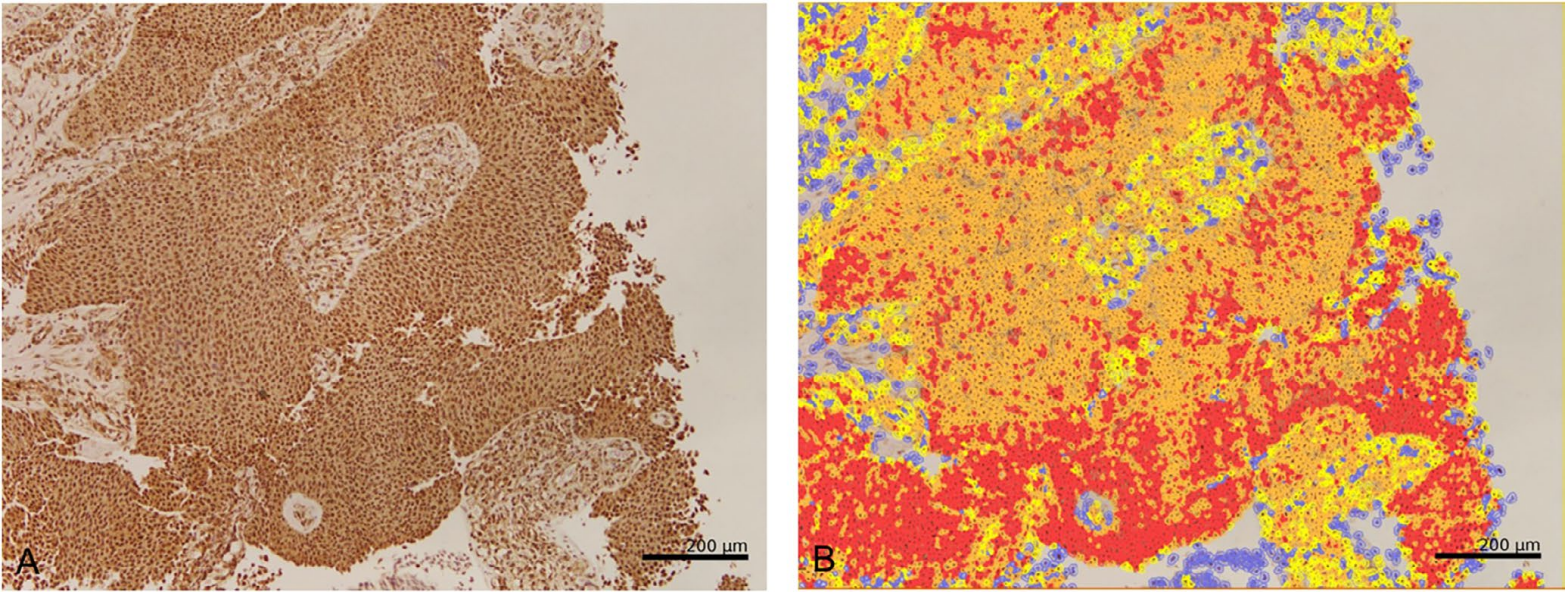
Representative phosphorylated LATS1/2 (p‐LATS1/2) immunohistochemistry and digital image assessment. Staining detects LATS1 phosphorylated at Thr1079 and LATS2 phosphorylated at Thr1041. (A) Immunohistochemical staining. (B) Digital staining‐intensity classification overlay. Scale bars, 200 μm.

**TABLE 1:** Clinicopathological characteristics by original p‐LATS1/2 group.

Characteristic	Total (*n* = 96)	Low (*n* = 48)	High (*n* = 48)	*p*
Age at RC (years)	71.5 (42.0–86.0)	73.0 (42.0–85.0)	70.0 (50.0–86.0)	0.200
BMI (kg/m^2^)	22.7 (14.8–31.6)	23.5 (15.1–30.2)	22.3 (14.8–31.6)	0.072
Female sex	17 (17.7)	14 (29.2)	3 (6.3)	0.006
Smoking history	60 (62.5)	25 (52.1)	35 (72.9)	0.057
Performance status ≥ 2	11 (11.5)	7 (14.6)	4 (8.3)	0.523
Neoadjuvant chemotherapy	62 (64.6)	36 (75.0)	26 (54.2)	0.054
Clinical T2 (including T2a)	52 (54.2)	21 (43.8)	31 (64.6)	0.097
Clinical T3	37 (38.5)	22 (45.8)	15 (31.3)	
Clinical T4	7 (7.3)	5 (10.4)	2 (4.2)	
RC p/ypT0–2	65 (67.7)	25 (52.1)	40 (83.3)	0.002
RC p/ypT3–4	31 (32.3)	23 (47.9)	8 (16.7)	
RC p/ypN0	83 (86.5)	38 (79.2)	45 (93.8)	0.070
RC p/ypN+	13 (13.5)	10 (20.8)	3 (6.3)	
High grade at baseline	87 (90.6)	43 (89.6)	44 (91.7)	1.000
Variant histology at baseline	21 (21.9)	11 (22.9)	10 (20.8)	1.000
Variant histology at RC	25 (26.0)	15 (31.3)	10 (20.8)	0.352
RC LVI, positive	36 (37.5)	21 (43.8)	15 (31.3)	0.208
RC LVI, missing	1 (1.0)	1 (2.1)	0 (0.0)	—
RC surgical margin, positive	10 (10.4)	8 (16.7)	2 (4.2)	0.051
RC surgical margin, missing	1 (1.0)	1 (2.1)	0 (0.0)	—
p‐LATS1/2 *H*‐score	77.35 (0.02–292.54)	17.01 (0.02–75.61)	228.89 (79.09–292.54)	—
Ki‐67 labeling index (%)	47.7 (9.0–94.0)	41.1 (9.0–91.6)	52.6 (13.1–94.0)	0.119
Nuclear YAP *H*‐score	104.7 ± 60.1	120.4 ± 56.7	88.9 ± 59.8	0.009
Microvessel density (MVD)	40.4 ± 19.0	38.1 ± 19.6	42.6 ± 18.3	0.245
Lymphatic vessel density (LVD)	10.9 ± 8.1	11.2 ± 8.3	10.7 ± 8.0	0.752

*Note:* Values are *n* (%) or median (range), except YAP, MVD and LVD, which are mean ± SD. Percentages use the full group denominators; missing values were excluded from statistical comparisons.

Abbreviations: BMI, body mass index; LVD, lymphatic vessel density; LVI, lymphovascular invasion; MVD, microvessel density; p‐LATS1/2, phosphorylated large tumor suppressor kinases 1 and 2; RC, radical cystectomy; SD, standard deviation; YAP, Yes‐associated protein.

Exploratory nuclear YAP assessment included all 96 patients (48 per p‐LATS1/2 group). *H*‐scores were higher in the low‐staining group (mean ± standard deviation, 120.4 ± 56.7 versus 88.9 ± 59.8; Mann–Whitney U *p* = 0.009).

### 3.2 Survival Outcomes

There were 43 deaths and 47 PFS events. Median follow‐up estimated by reverse Kaplan–Meier was 67 months. Median OS was 53 versus 104 months in the low‐ versus high‐staining groups (log‐rank *p* = 0.039); median PFS was 24 versus 90 months (*p* = 0.051; Figure 2).

**FIGURE 2:**
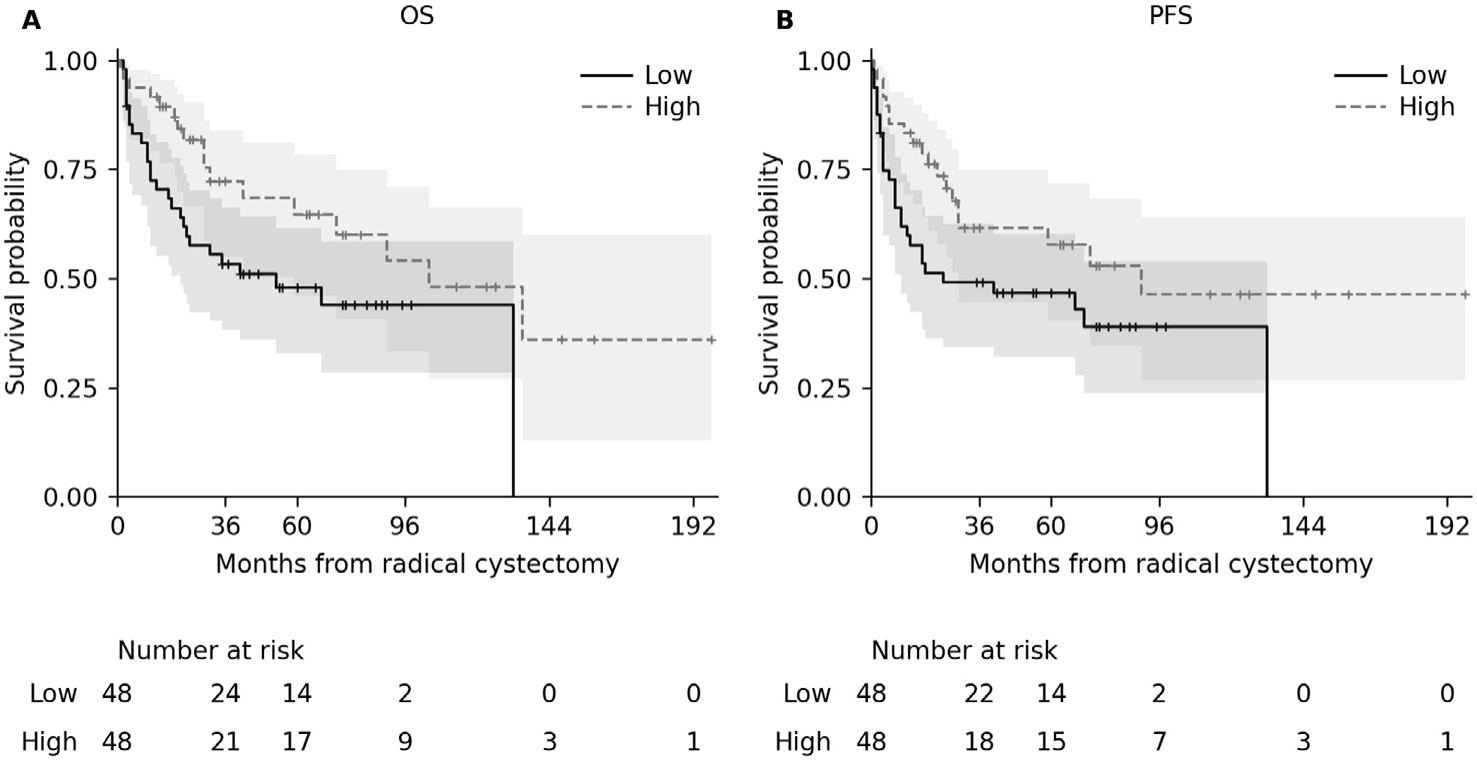
Kaplan–Meier estimates of (A) overall survival (OS) and (B) progression‐free survival (PFS) from radical cystectomy by phosphorylated large tumor suppressor kinases 1 and 2 (p‐LATS1/2) staining group (48 patients per group). Black solid and gray dashed lines indicate the low‐ and high‐staining groups, respectively. Shading indicates pointwise 95% confidence intervals; plus signs indicate censoring. Numbers at risk immediately before the indicated times are shown below each panel. Log‐rank *p* values were 0.039 for OS and 0.051 for PFS. PFS was defined by the first recorded metastasis or all‐cause death.

In extended models (*n* = 95), each 10‐point increase in p‐LATS1/2 *H*‐score was associated with lower mortality (HR, 0.956; 95% CI: 0.920–0.993; *p* = 0.020) and PFS‐event risk (0.962; 0.929–0.996; *p* = 0.029). Corresponding base‐model estimates for all 96 patients were 0.963 (0.927–0.999; *p* = 0.045) and 0.967 (0.935–1.001; *p* = 0.054). Comparisons using the same 95 patients appear in Tables 2 and 3. Extended‐model low‐versus‐high associations were not statistically significant for OS (HR, 1.777; 95% CI: 0.871–3.624; *p* = 0.114) and PFS (1.718; 0.872–3.386; *p* = 0.118).

**TABLE 2:** Multivariable Cox proportional hazards models for overall survival.

Variable	Continuous p‐LATS1/2 (per 10 *H*‐score points)			Low/high p‐LATS1/2 (low vs high)		
	HR	95% CI	*p*	HR	95% CI	*p*
Base model (*n* = 96)						
Age (per year)	1.031	0.989–1.074	0.153	1.034	0.991–1.079	0.118
p/ypT3–4 vs. p/ypT0–2	2.731	1.296–5.754	0.008	2.824	1.336–5.971	0.007
p/ypN+ vs. p/ypN0	1.243	0.551–2.804	0.600	1.313	0.582–2.965	0.512
p‐LATS1/2	0.963	0.927–0.999	0.045	1.511	0.739–3.089	0.258
Ki‐67 (per percentage point)	1.025	1.007–1.043	0.006	1.022	1.004–1.039	0.014
Base model, complete cases (*n* = 95)						
Age (per year)	1.033	0.991–1.077	0.126	1.036	0.994–1.081	0.097
p/ypT3–4 vs. p/ypT0–2	2.574	1.219–5.436	0.013	2.631	1.237–5.596	0.012
p/ypN+ vs. p/ypN0	1.234	0.548–2.779	0.611	1.293	0.574–2.916	0.535
p‐LATS1/2	0.961	0.926–0.998	0.038	1.605	0.783–3.290	0.196
Ki‐67 (per percentage point)	1.025	1.008–1.043	0.005	1.022	1.005–1.040	0.011
Extended model, complete cases (*n* = 95)						
Age (per year)	1.026	0.982–1.072	0.252	1.030	0.986–1.077	0.180
p/ypT3–4 vs. p/ypT0–2	2.044	0.823–5.077	0.124	1.978	0.767–5.101	0.158
p/ypN+ vs. p/ypN0	0.877	0.353–2.177	0.778	0.935	0.373–2.344	0.885
p‐LATS1/2	0.956	0.920–0.993	0.020	1.777	0.871–3.624	0.114
Ki‐67 (per percentage point)	1.030	1.012–1.049	< 0.001	1.027	1.010–1.046	0.002
RC LVI (positive vs. negative)	1.113	0.513–2.415	0.787	1.207	0.540–2.702	0.646
RC margin (positive vs. negative)	3.830	1.455–10.082	0.007	3.562	1.342–9.456	0.011

*Note:* Continuous and categorical p‐LATS1/2 were evaluated in separate models. All models included 43 deaths. Both *n* = 95 analyses used the same complete‐case cohort, excluding one patient with missing LVI and surgical margin data. The prefixes *p* and yp denote pathological staging without and after neoadjuvant therapy, respectively; *T* and *N* denote primary tumor and regional lymph node categories.

Abbreviations: CI, confidence interval; HR, hazard ratio; LVI, lymphovascular invasion; p‐LATS1/2, phosphorylated large tumor suppressor kinases 1 and 2; RC, radical cystectomy.

**TABLE 3:** Multivariable Cox proportional hazards models for progression‐free survival.

Variable	Continuous p‐LATS1/2 (per 10 *H*‐score points)			Low/high p‐LATS1/2 (low vs. high)		
	HR	95% CI	*p*	HR	95% CI	*p*
Base model (*n* = 96)						
Age (per year)	1.012	0.973–1.052	0.563	1.014	0.974–1.054	0.500
p/ypT3–4 vs. p/ypT0–2	2.966	1.493–5.895	0.002	3.044	1.529–6.063	0.002
p/ypN+ vs. p/ypN0	1.463	0.657–3.259	0.352	1.581	0.710–3.522	0.262
p‐LATS1/2	0.967	0.935–1.001	0.054	1.449	0.737–2.849	0.282
Ki‐67 (per percentage point)	1.019	1.002–1.035	0.025	1.016	1.000–1.032	0.057
Base model, complete cases (*n* = 95)						
Age (per year)	1.014	0.975–1.054	0.496	1.016	0.976–1.057	0.440
p/ypT3–4 vs. p/ypT0–2	2.829	1.421–5.632	0.003	2.881	1.440–5.761	0.003
p/ypN+ vs. p/ypN0	1.441	0.648–3.204	0.370	1.543	0.694–3.433	0.287
p‐LATS1/2	0.966	0.934–0.999	0.045	1.531	0.777–3.017	0.219
Ki‐67 (per percentage point)	1.019	1.003–1.036	0.021	1.016	1.000–1.033	0.044
Extended model, complete cases (*n* = 95)						
Age (per year)	1.002	0.961–1.044	0.940	1.004	0.964–1.046	0.842
p/ypT3–4 vs. p/ypT0–2	1.841	0.817–4.148	0.141	1.727	0.741–4.025	0.206
p/ypN+ vs. p/ypN0	1.352	0.567–3.222	0.496	1.481	0.622–3.522	0.375
p‐LATS1/2	0.962	0.929–0.996	0.029	1.718	0.872–3.386	0.118
Ki‐67 (per percentage point)	1.023	1.007–1.040	0.006	1.020	1.004–1.037	0.013
RC LVI (positive vs. negative)	1.735	0.859–3.505	0.125	1.884	0.904–3.929	0.091
RC margin (positive vs. negative)	3.233	1.277–8.181	0.013	3.059	1.197–7.818	0.019

*Note:* Continuous and categorical p‐LATS1/2 were evaluated in separate models. All models included 47 PFS events. Both *n* = 95 analyses used the same complete‐case cohort, excluding one patient with missing LVI and surgical margin data. PFS was defined as the time from RC to the first recorded metastasis or all‐cause death; local‐recurrence‐only events were not captured. The prefixes *p* and yp denote pathological staging without and after neoadjuvant therapy, respectively; *T* and *N* denote primary tumor and regional lymph node categories.

Abbreviations: CI, confidence interval; HR, hazard ratio; LVI, lymphovascular invasion; p‐LATS1/2, phosphorylated large tumor suppressor kinases 1 and 2; PFS, progression‐free survival; RC, radical cystectomy.

All specified Cox models converged. Proportional‐hazards diagnostics suggested a possible violation for pathological *T* in the base continuous OS model (*n* = 96; *p* = 0.030; global *p* = 0.273). Extended continuous OS model diagnostics yielded global *p* = 0.428 and p‐LATS1/2‐specific *p* = 0.264. Leave‐one‐out p‐LATS1/2 *p* values ranged from 0.007–0.053 for OS and 0.014–0.072 for PFS. No patient was excluded based on significance. Excluding the discordant‐LVI case yielded similar OS estimates (Table S3).

### 3.3 Exploratory Analyses by Chemotherapy Status

Without NAC, low/high log‐rank *p* values were 0.002 for OS and 0.025 for PFS; with NAC, they were 0.631 and 0.283, respectively (Figure 3). OS interaction tests were nonsignificant (continuous *p* = 0.712; binary *p* = 0.431; Table 4).

**FIGURE 3:**
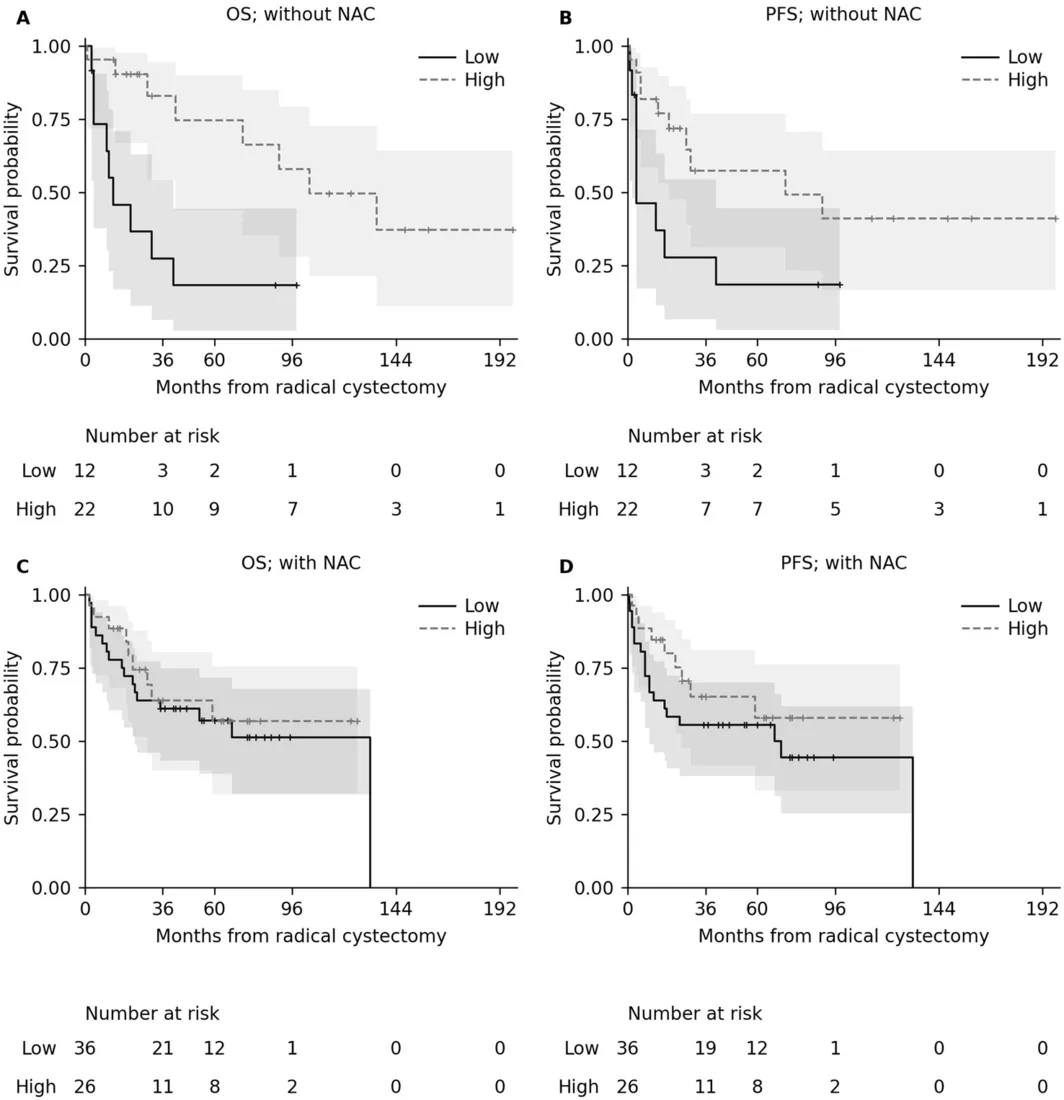
Kaplan–Meier estimates of overall survival (OS) and progression‐free survival (PFS) from radical cystectomy according to neoadjuvant chemotherapy (NAC) status and phosphorylated large tumor suppressor kinases 1 and 2 (p‐LATS1/2) staining group. (A, B) Patients without NAC (12 low‐ and 22 high‐staining patients); (C, D) patients with NAC (36 low‐ and 26 high‐staining patients). OS is shown in A and C and PFS in B and D. Black solid and gray dashed lines indicate the low‐ and high‐staining groups, respectively. Shading indicates pointwise 95% confidence intervals; plus signs indicate censoring. Numbers at risk immediately before the indicated times are shown below each panel. Log‐rank *p* values were 0.002, 0.025, 0.631 and 0.283 for A–D, respectively. PFS was defined by the first recorded metastasis or all‐cause death.

**TABLE 4:** Exploratory p‐LATS1/2 by NAC interaction models for overall survival.

Variable	Continuous p‐LATS1/2 (per 1 *H*‐score point)			Low/high p‐LATS1/2 (low vs high)		
	HR	95% CI	*p*	HR	95% CI	*p*
Age (per year)	1.028	0.986–1.072	0.197	1.031	0.988–1.077	0.162
p/ypT3–4 vs. p/ypT0–2	2.574	1.201–5.517	0.015	2.618	1.213–5.649	0.014
p/ypN+ vs. p/ypN0	1.475	0.592–3.673	0.404	1.590	0.637–3.966	0.320
p‐LATS1/2	0.995	0.990–1.001	0.085	2.163	0.729–6.423	0.165
Ki‐67 (per percentage point)	1.024	1.006–1.042	0.009	1.020	1.002–1.038	0.027
NAC (yes vs. no)	0.670	0.250–1.794	0.425	1.083	0.379–3.091	0.882
p‐LATS1/2 × NAC	1.001	0.994–1.009	0.712	0.570	0.141–2.309	0.431

*Note:* Both models included 96 patients and 43 deaths. The p‐LATS1/2 main effect applies to patients without NAC. The NAC main effect applies at *H*‐score 0 in the continuous model and in the high p‐LATS1/2 group in the categorical model. The prefixes *p* and yp denote pathological staging without and after neoadjuvant therapy, respectively; *T* and *N* denote primary tumor and regional lymph node categories.

Abbreviations: CI, confidence interval; HR, hazard ratio; NAC, neoadjuvant chemotherapy; p‐LATS1/2, phosphorylated large tumor suppressor kinases 1 and 2.

### 3.4 Matching and Exploratory Cutoff Analyses

Matching yielded 30 pairs with 24 deaths and 27 PFS events. Absolute standardized mean differences for age and clinical nodal status remained 0.181 and 0.150, failing the specified balance criterion; matched survival inference was therefore omitted (Table S1; Figure S1).

The 36‐month AUC was 0.615 (95% CI: 0.485–0.739). The exploratory cutoff was 45.006 (lower/higher groups: 38/58 patients). Apparent sensitivity/specificity of 0.593/0.628 decreased to 0.541/0.594 after optimism correction. Bootstrap cutoff interquartile and central 95% ranges were 28.297–187.664 and 7.125–202.279, respectively. No selected‐cutoff survival test was performed (Table S2; Figure S2).

### 3.5 Paired TUR and RC Specimens

Paired assessment included 71/96 patients (41 receiving NAC); 25 lacked evaluable RC tissue because of ypT0 or unavailable specimens. TUR and RC scores correlated (Spearman's *ρ* = 0.520; *p* < 0.001), with categorical agreement in 54/71 (76.1%). Among NAC‐naïve patients, 30/34 had paired specimens; *ρ* was 0.615 (*p* < 0.001), with agreement in 25/30 (83.3%; Figure 4).

**FIGURE 4:**
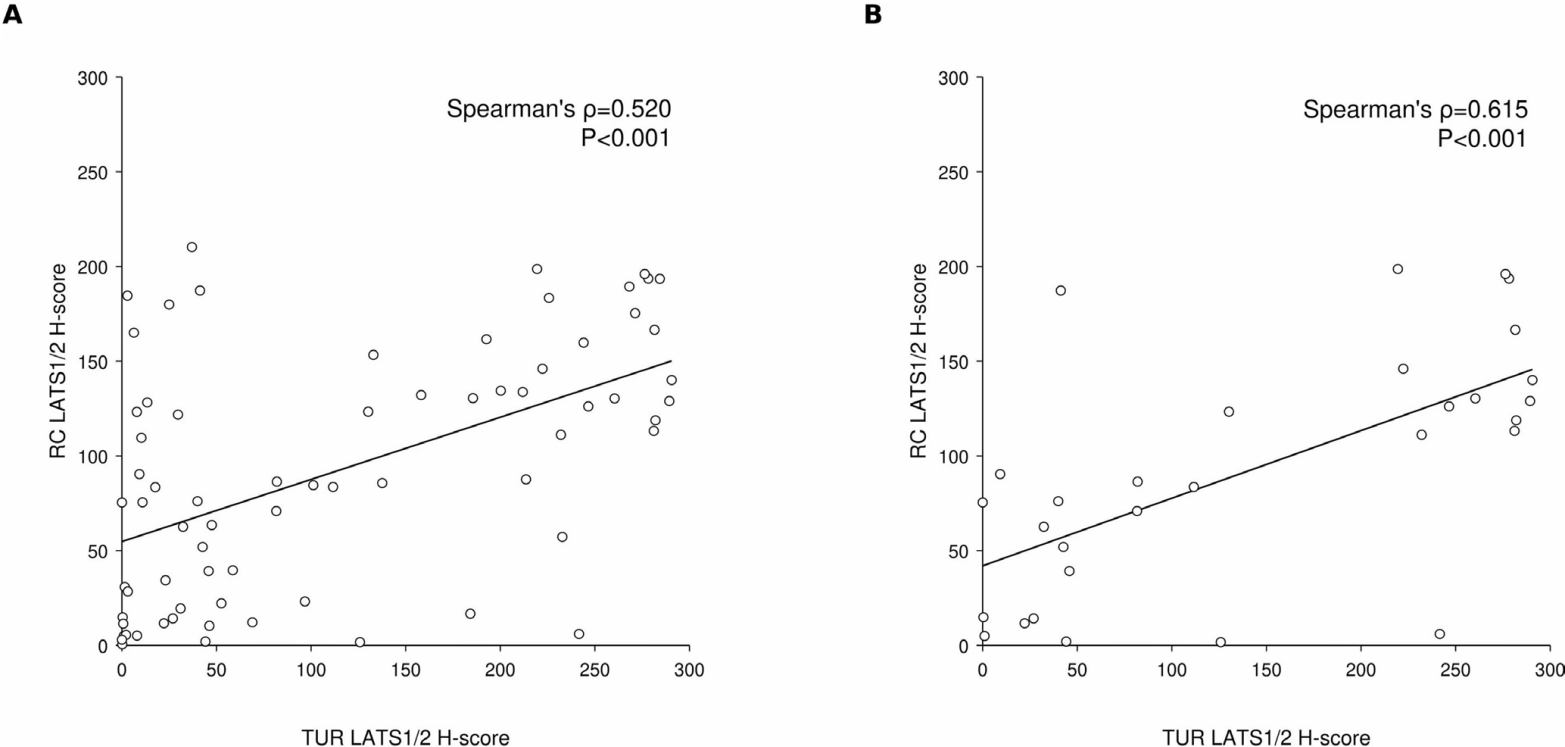
Phosphorylated LATS1/2 (p‐LATS1/2) *H*‐scores in paired transurethral resection (TUR) and radical cystectomy (RC) specimens. (A) Overall paired cohort (*n* = 71; Spearman's *ρ* = 0.520; *p* < 0.001). (B) Patients without neoadjuvant chemotherapy (*n* = 30; Spearman's ρ = 0.615; *p* < 0.001). The LATS1/2 *H*‐score labels in the author‐supplied panels refer to p‐LATS1/2 immunoreactivity. Solid lines indicate fitted linear regression lines.

## 4 Discussion

In this RC cohort, lower continuous p‐LATS1/2 *H*‐scores were associated with shorter OS and PFS after adjustment for selected pathological variables, including LVI and margin status. This finding supports further evaluation of a quantitative prognostic tissue marker. Large cystectomy studies establish the prognostic importance of LVI and soft‐tissue margins [3, 4]; the greater prevalence of advanced RC stage among low‐staining tumors also underscores the importance of pathological adjustment. Nevertheless, residual confounding remains possible. Binary‐group confidence intervals were wide and included the null. Dichotomization discards within‐group information, while limited event counts constrain precision. The continuous association therefore does not validate the median‐defined classification or establish pretreatment applicability.

Higher nuclear YAP in low‐p‐LATS1/2 tumors was consistent with canonical LATS‐mediated restraint [6, 7, 8]. Interpretation requires attention to assay specificity: phospho‐specific staining measures neither total LATS abundance nor catalytic activity and cannot distinguish LATS1 from LATS2. Without total‐LATS measurements, lower staining could reflect reduced protein abundance or a reduced phosphorylated fraction. NUAK2 experiments concern inhibitory LATS sites distinct from the activation‐associated sites detected here [13]. Clinical YAP evidence is heterogeneous: positive findings in 213 urothelial tumors contrast with negative compartment‐specific findings in a cohort comprising 192 muscle‐invasive and 192 non‐muscle‐invasive tumors [11, 12]. Differences in analyte, compartment, scoring and populations may contribute, although their roles remain unproven. Studies of WWC1 in upper tract urothelial carcinoma and total LATS2 in prostate cancer [22, 23] further illustrate the need for assay‐specific interpretation. Our cross‐sectional YAP finding supports biological plausibility but does not establish transcriptional activity or mediation of survival, nor validate p‐LATS1/2 as a surrogate of pathway output. Direct assessment of phosphorylated YAP, TAZ and transcriptional targets is needed. Vascular density and Ki‐67 cannot establish a separate mechanism; immune infiltration, checkpoint expression and genomic instability were not assessed.

Experimental evidence linking YAP to cisplatin resistance [9] motivates investigation of treatment‐related associations. However, formal OS interactions with NAC were nonsignificant, and differences between subgroup *p* values do not demonstrate chemotherapy benefit or effect modification. The specified matching approach left residual imbalance in age and clinical nodal status and retained fewer events, precluding matched survival inference with this design. Because NAC follows TUR measurement, comparisons conditional on treatment history concern survival from the RC landmark and cannot establish a causal pretreatment biomarker effect.

Paired TUR–RC observations demonstrate measurement feasibility, although moderate correlations and incomplete agreement limit specimen interchangeability. NAC‐naïve pairs reduce one source of variation. Published paired‐bladder studies likewise show biomarker‐specific concordance and treatment‐associated changes [24, 25], while our data cannot distinguish sampling, technical, and treatment contributions. Restriction to evaluable residual RC tumor also excludes complete responders. Selected TUR fields included representative invasive fronts, but diagnostic sections did not consistently permit delineation of both tumor‐core and invasive‐front regions. Accordingly, *H*‐scores describe sampled‐field immunoreactivity, without a separately annotated regional comparison; TUR–RC agreement does not characterize within‐tumor spatial heterogeneity. Distinct immunophenotypic molecular subtypes can occur in different histological regions of bladder tumors [26]; whether p‐LATS1/2 varies spatially requires investigation with predefined compartments and sampling criteria.

The next clinical question is whether p‐LATS1/2 adds predictive information to established clinicopathological models. RC nomograms [5] and prospective multi‐marker immunohistochemical studies [27] provide relevant comparators. The international LVI study found only small overall improvements in predictive accuracy despite prognostic associations [3], whereas a prospective cell‐cycle/proliferation‐marker panel increased a cancer‐specific mortality model's concordance index from 0.80 to 0.83 [27]. Our marginal 36‐month AUC describes the staining score alone; it does not measure incremental discrimination, calibration or clinical benefit and is not directly comparable with concordance indices for different outcomes, populations or multivariable models. Modest discrimination, widely dispersed bootstrap cutoffs, and reduced optimism‐corrected performance preclude adoption of the selected threshold. Resampling evaluates internal instability rather than external validity, and marginal censoring weights assume independent censoring [20]. Continuous scores and the median‐defined grouping remained the primary analyses.

This single‐center retrospective study included 96 patients selected for RC. Seven‐coefficient models were supported by 43 deaths and 47 PFS events, with only 10 positive margins. Possible departures from proportional hazards for pathological T stage and leave‐one‐out sensitivity warrant caution despite numerical convergence. The principal Cox models omitted sex, clinical stage, and NAC, leaving potential confounding from these factors and treatment selection. One early metastasis interval was approximate, and the operational PFS endpoint may omit local‐recurrence‐only events. Functional experiments, molecular profiling, and independent public‐dataset validation were not performed.

Future validation should build on the continuous information and explicit thresholds afforded by digital assessment [16]. A multicenter automated Ki‐67 study demonstrated the importance of scoring strategy and imaging workflow for reproducibility [28]; fixed QuPath settings alone do not validate this cytoplasmic phospho‐specific assay. Delayed fixation can alter phosphoepitopes in a marker‐dependent manner in breast tissue [29], although susceptibility of bladder p‐LATS1/2 remains untested. Multicenter studies should standardize tissue handling and sampling, quantify observer/laboratory agreement, and prespecify selection, assays, endpoints, marker coding and missing‐data handling following REMARK recommendations [30]. Independent evaluation should compare models with and without p‐LATS1/2, assess calibration and discrimination, and fix any threshold beforehand. Covariates must match the intended decision time: pretreatment use requires pretreatment predictors, whereas adding TUR staining to RC pathology addresses postoperative prognosis. Treatment‐guiding use requires evidence that assay‐based decisions improve clinical outcomes.

In conclusion, lower continuous p‐LATS1/2 *H*‐scores were associated with shorter OS after RC, whereas binary‐group estimates remained imprecise. These findings support further investigation of p‐LATS1/2 as a candidate prognostic marker, with independent clinical and functional validation required before clinical application.

## Author Contributions

**Shota Kakita:** methodology, investigation, data curation, validation, writing – original draft, writing – review and editing. **Tomohiro Matsuo:** conceptualization, methodology, data curation, formal analysis, visualization, project administration, writing – original draft, writing – review and editing, validation. **Asato Otsubo:** data curation, writing – review and editing. **Kyohei Araki:** data curation, writing – review and editing. **Hiroyuki Honda:** investigation. **Shintaro Mori:** data curation, investigation, writing – review and editing. **Kensuke Mitsunari:** writing – review and editing. **Kojiro Ohba:** writing – review and editing. **Ryoichi Imamura:** methodology, supervision, writing – review and editing.

## Funding

This work was supported by JSPS KAKENHI Grant Number JP23K08760.

## Ethics Statement

The study protocol was approved by the Institutional Review Board of Nagasaki University Hospital (No. 2312815).

## Consent

The requirement for written informed consent was waived by the Institutional Review Board of Nagasaki University Hospital because of the retrospective nature of the study, and an opt‐out procedure was implemented.

## Conflicts of Interest

The authors declare no conflicts of interest.

## Supporting information


**Figure S1:** Propensity‐score matching and covariate balance. (A) Absolute standardized mean differences (SMDs) before and after matching, calculated using fixed pre‐matching denominators. The dashed vertical line indicates an SMD of 0.10; the horizontal line separates the five propensity‐score covariates from additional descriptive covariates. (B) Propensity‐score distributions before and after matching; dotted lines indicate the common‐support limits. Low and high denote the original median‐defined p‐LATS1/2 groups. Matching retained 30 pairs; age and clinical nodal status remained above the balance threshold, and matched survival analyses were not performed. BMI, body mass index; cN, clinical nodal category; cT, clinical T category; ECOG, Eastern Cooperative Oncology Group performance status; NAC, neoadjuvant chemotherapy; p‐LATS1/2, phosphorylated large tumor suppressor kinases 1 and 2.

**Figure S2:** Exploratory 36‐month overall survival analysis from radical cystectomy in 96 patients. (A) Cumulative/dynamic receiver operating characteristic curve using inverse‐probability‐of‐censoring weighting. The diagonal indicates chance discrimination; the point represents the selected cutoff. (B) Youden‐index cutoff search (left) and distribution of selected cutoffs across 2000 bootstrap resamples (right). Solid vertical lines indicate the original selected cutoff of 45.006; the dashed line indicates the original median. Cutoff selection required at least 20 patients per group and was repeated with censoring estimation in every resample. Lower scores indicate higher risk. (C) Apparent and bootstrap optimism‐corrected performance at the selected cutoff. Bootstrap variability reflects cutoff instability, not a clinically validated threshold. AUC, area under the curve; CI, confidence interval; p‐LATS1/2, phosphorylated large tumor suppressor kinases 1 and 2.

**Table S1:** Propensity score matching balance and retention. Covariate balance and patient retention before and after matching.
**Table S2:** Exploratory 36‐month overall survival ROC analysis. Continuous‐marker discrimination, selected‐cutoff performance, and bootstrap cutoff stability. ROC, receiver operating characteristic.
**Table S3:** Model diagnostics and sensitivity summaries. Proportional‐hazards and nonlinearity diagnostics, leave‐one‐out analyses, and sensitivity analysis excluding the discordant lymphovascular invasion record.
Supplementary Methods. Detailed immunohistochemical staining and digital image analysis protocols, analysis population and endpoint derivation, statistical models and diagnostics, propensity‐score matching, time‐dependent receiver operating characteristic analysis, and computational verification.

## Data Availability

The data that support the findings of this study are available on request from the corresponding author. The data are not publicly available due to privacy or ethical restrictions.
