# Supplementary material for: Phospho‐LATS1/2 Immunoreactivity and Survival in Muscle‐Invasive Bladder Cancer: A Quantitative Immunohistochemical Study
Source: Int J Urol. 2026 Nov 13;33(11):e70715. doi: 10.1111/iju.70715 (PMC13768573; doi:10.1111/iju.70715)
Supplement: Supplementary file 1 [file IJU-33-0-s001.docx]

**Supplementary Methods**

**Immunohistochemical staining and digital image analysis**

Heat-induced retrieval used 0.01 M sodium citrate (pH 9.0), at 95°C for 40 minutes for p-LATS1/2, YAP, CD34 and D2-40, and 121°C for 15 minutes for Ki-67. Peroxidase was blocked with 3% hydrogen peroxide for 30 minutes. Overnight incubation at 4°C used p-LATS1/2 (Abcam, ab111344, 1:200), YAP (Cell Signaling Technology, D8H1X/14074, 1:400), Ki-67 (Dako, ready-to-use), CD34 (Dako, 1:50) and D2-40 (Dako, 1:200). The p-LATS1/2 antibody recognizes LATS1 phosphorylated at Thr1079 and LATS2 phosphorylated at Thr1041; the H-score therefore quantifies combined p-LATS1/2 immunoreactivity rather than total LATS1/2 abundance. EnVision+ HRP (Dako, 5007), diaminobenzidine and hematoxylin provided detection and counterstaining.

QuPath 0.5.1 quantified selected tumor fields, including representative invasive fronts. Nuclear detection used area 10–1000 px², threshold 0.1 and cell expansion 5 px. Cytoplasmic p-LATS1/2 and nuclear YAP intensity categories used optical-density thresholds 0.20, 0.25 and 0.30. The percentage of tumor cells in each staining-intensity category was automatically exported to calculate the H-score:

H-score = (1 × % weak) + (2 × % moderate) + (3 × % strong).

The same staining and scoring protocol was used for paired RC specimens. The main-text H-score and historical grouping definitions were retained unchanged.

**Analysis population and endpoint derivation**

All 96 patients were retained in the original cohort and original low/high p-LATS1/2 groups (48 each). Following author CT/MRI re-review, all 13 cases previously tabulated as clinical T1 were classified as cT2a; they were not excluded. Clinical staging was distinguished from pathological/yp staging. The source category labeled “T1is” was confirmed to belong to the p/ypT0–2 group and was classified accordingly.

Survival time was measured from radical cystectomy (RC). Overall survival (OS) used recorded time to all-cause death for deceased patients and recorded RC follow-up time for survivors. Recorded follow-up occurring after a recorded death did not extend OS. Progression-free survival (PFS) was reconstructed as the earliest flagged metastasis or all-cause death time; otherwise the last documented progression-free follow-up was used. Local-recurrence-only events were not added where not recorded. OS contained 43 deaths and 53 censored observations; PFS contained 47 events and 49 censored observations. The authors confirmed that the last progression-free assessment and last survival follow-up coincided in the four queried event-free records. The patient whose metastasis was initially recorded at 0 months had no metastasis at RC; metastasis was identified approximately 20 days later. This approximate interval was analyzed as 20/(365.25/12) = 0.657084 months, without implying exact-date precision. Correcting this first event time did not change event order or risk sets, and the repeated Cox estimates, diagnostics and log-rank statistics were unchanged. Reconstructed PFS did not exceed OS.

**Baseline group comparisons**

Between-group comparisons used the Mann–Whitney U test for age, BMI, Ki-67 and nuclear YAP, Student’s t test for microvessel density and lymphatic vessel density, and Fisher’s exact test for categorical variables. Corrected clinical T categories (2 including 2a, 3 and 4) were compared using the Fisher–Freeman–Halton exact test.

**Cox proportional hazards models**

The base multivariable specification contained age per 1 year, p/ypT3–4 versus 0–2, p/ypN-positive versus N0, continuous p-LATS1/2 H-score per 1 point, and Ki-67 per 1 percentage point. The extended specification added binary RC lymphovascular invasion (LVI) and surgical margin status. One patient lacked both additional variables; extended models therefore used 95 complete cases, retaining all 43 OS and 47 PFS events. Base models were shown both in all 96 and in the identical 95-case set used for the extension. No missing value was converted to negative and no imputation was used.

Binary models replaced continuous p-LATS1/2 with the original low/high classification, with high as reference. The grouping was fixed before complete-case restriction. No patient lay at the reported median threshold 77; the reported value and the median of approximately 77.352 produced identical original membership. Both marker representations were not included simultaneously. Per-10-point continuous-marker HRs are algebraic rescalings of the per-1-point coefficient, not separate models.

Cox models used Efron handling of tied event times and risk sets including patients censored at that same time. Model-based covariance was the inverse observed information. HRs, two-sided Wald P values and 95% Wald confidence intervals were calculated. There was no univariable screening, stepwise selection, optimized cutoff, or penalization in the primary models. The base covariate set was recovered from the submitted tables; an independently accessible preregistration was not available.

The original exploratory OS interaction specification added NAC and p-LATS1/2-by-NAC interaction to the five base covariates, retaining both main effects. Continuous and original-binary versions were fitted. No new PFS interaction or LVI/margin-extended interaction model was introduced. With an uncentered continuous marker, the NAC main coefficient is conditional on H-score 0; it is not an average causal treatment effect. Differing subgroup P values were not treated as evidence of interaction.

**Model diagnostics**

Convergence, score norms, positive information eigenvalues and information conditioning were checked. Proportional hazards were examined using efficient score tests for time-dependent coefficients with a centered 1 − KM(t−) transform. The covariance of the added time effects was adjusted for the fitted constant coefficients through the nuisance-information Schur complement. Individual one-degree-of-freedom and joint global tests were reported, together with Efron-adjusted scaled Schoenfeld plots. A nonsignificant global test was not used to disregard a covariate-specific signal or to prove proportional hazards.

For continuous variables, efficient score tests assessed an additional restricted-cubic-spline nonlinear term with design-based knots at the 10th, 50th and 90th percentiles. These were diagnostics, not substituted primary fits. Exact leave-one-out refits assessed influence; no observation was excluded from the primary analyses because of its influence or resulting P value. A separately labeled sensitivity excluded the single discordant total-LVI record; the reported total LVI value was retained in the primary analysis. Finite convergence, nonsignificant assumption tests and stable effect direction were not interpreted as eliminating overfitting or sparse-data uncertainty.

**Descriptive survival**

Kaplan-Meier estimates used RC-origin endpoints, pointwise log-log confidence intervals, censoring marks and numbers at risk immediately before displayed times. Full-cohort and original NAC-stratified low/high comparisons were descriptive; unadjusted logrank comparisons used the hypergeometric tied-event variance. Reverse Kaplan-Meier follow-up used deaths before censoring at tied original recorded times. Sparse tail estimates were explicitly qualified. Secondary P values were not multiplicity adjusted.

**Fixed propensity-score matching**

Using specifications fixed during this revision before examining matching results, an unpenalized logistic model estimated the probability of original low-p-LATS1/2 membership from age, female sex, author-corrected pre-NAC cT3–4 versus cT2/cT2a, clinical N-positive versus N0, and NAC receipt. These variables were complete in all 96 patients. H-score, survival outcomes and postoperative pathological factors were not predictors. Because NAC follows marker measurement but precedes RC, the target is a prognostic comparison at the RC landmark conditional on treatment history, not solely pretreatment causal adjustment.

One-to-one nearest-neighbor matching on the fitted logit used no replacement and a caliper of 0.2 times the pre-match pooled within-group sample SD of the logit. Common support was the intersection of the two group ranges. The score was not re-estimated after restriction. Low-group patients were processed in descending score order; deterministic identifier ordering resolved numerical ties. No score covariate, caliper, ratio, order or replacement setting was retuned after examining matching results.

Balance used standardized mean differences (SMDs) with fixed pre-match denominators. For continuous variables the denominator was the square root of the mean of the two pre-match sample variances; for binary variables it was the square root of the mean Bernoulli variance. All five modeled absolute SMDs had to be below 0.10. The frozen match produced 30 pairs but age and clinical N remained above this threshold. The stop condition fixed during this revision was therefore applied: matched inferential Cox/logrank analyses were not run. Patient/event retention, overlap and balance were reported descriptively. No claim was made that matching eliminated measured or unmeasured confounding.

**Exploratory 36 month ROC and cutoff stability**

The approved single horizon was 36-month all-cause OS after RC, with lower p-LATS1/2 fixed as higher risk before the revision analysis. Cumulative/dynamic ROC used inverse-probability-of-censoring weights from a marginal reverse Kaplan-Meier model. At tied recorded times deaths preceded censoring, giving censoring hazard c/(Y-d). Cases were deaths at or before 36 months and controls had observed survival beyond 36 months. Case weights were 1/G(T−); control weights were 1/G(36). Early-censored patients remained in the censoring fit and group-size counts. Sensitivity and specificity were normalized within their weighted case/control groups. AUC used the weighted ROC with half credit for marker ties.

Candidate thresholds were midpoints of consecutive distinct H-scores, restricted to at least 20 observations in each derivation group. Youden index was maximized; numerical ties were resolved by proximity to the original sample median, then the smaller threshold. In the earlier Python analyses, a 2,000-replicate patient bootstrap (NumPy PCG64 seed 20261005) repeated censoring estimation, candidate construction, group-size restriction and threshold selection in every resample. Duplicate draws counted as observations. No resample was discarded for an unfavorable threshold or performance estimate.

Each bootstrap-selected threshold was evaluated in its derivation resample with the resample censoring model and in the original cohort with the original censoring model. The mean difference was estimated optimism and was subtracted from the original apparent sensitivity, specificity and Youden index. Threshold selection frequencies and percentile ranges summarized instability. These ranges were not described as validated threshold confidence intervals or calibrated intervals for optimism-corrected selected-threshold performance. The continuous-marker AUC received a patient-bootstrap percentile 95% interval, with censoring re-estimated in every resample. No naive post-selection cutoff-based Cox P value or confirmatory clinical threshold was reported.

**Software reproducibility and verification**

The final survival analyses were performed using EZR version 1.68. The final group-comparison P values in Table 1 were calculated using EZR version 1.68. Earlier computational analyses and verification were conducted using Python 3.12.14, NumPy 2.3.5, SciPy 1.17.0, pandas 2.2.3 and matplotlib 3.10.8. The earlier Python Cox implementation used analytic Efron likelihood, score and information. Independently coded numerical likelihood/derivative calculations reproduced all 12 primary fits and both original OS interactions. Separate time-dependent likelihood calculations reproduced key PH tests. Matching pairs and balance were independently reproduced. Independently coded IPCW/ROC calculations reproduced the apparent values, all 2,000 resamples and all 71,744 admissible bootstrap candidate rows. Synthetic tie, horizon, zero-time, weighted-mass, time-translation and ROC integration checks were used. A full separate-directory rerun reproduced all 14 Cox coefficient/SE vectors, 30 pairs and 2,000 bootstrap outputs exactly. All 14 Cox fits were additionally verified using statsmodels 0.14.5 PHReg with Efron ties and model-based covariance; coefficients, standard errors, HRs, 95% CIs and two-sided Wald P values agreed. SciPy 1.17.0 independently reproduced all six log-rank tests and the 12 Kaplan–Meier curves at the checked times, including confidence intervals and numbers at risk.

These earlier Python checks establish numerical reproducibility of the stated computations, not the correctness of unverified clinical dates, independent censoring, causal validity, adequate sample size or external clinical validity. The earlier Python PH, nonlinearity, influence and time-dependent ROC analyses were not additionally verified with separate standard-software engines.

Matching and time-dependent ROC methods are supported by Austin (2011) and Blanche et al. (2013), cited as references 19 and 20 in the main manuscript.

Table S1 Propensity score matching balance and retention

| **Covariate** | **Before Low n = 48** | **Before High n = 48** | **After Low n = 30** | **After High n = 30** | **\|SMD\| Before** | **\|SMD\| After** |
| --- | --- | --- | --- | --- | --- | --- |
| Age in years | 72.0 (9.6) | 70.3 (7.6) | 71.4 (9.3) | 69.8 (7.1) | 0.202 | 0.181 |
| Female sex | 14 (29.2) | 3 (6.3) | 2 (6.7) | 3 (10.0) | 0.629 | 0.092 |
| Clinical T3–4 | 27 (56.3) | 17 (35.4) | 14 (46.7) | 14 (46.7) | 0.428 | 0.000 |
| Clinical N+ | 3 (6.3) | 2 (4.2) | 1 (3.3) | 2 (6.7) | 0.094 | 0.150 |
| NAC | 36 (75.0) | 26 (54.2) | 24 (80.0) | 23 (76.7) | 0.446 | 0.071 |
| BMI in kg/m² | 22.9 (3.2) | 21.9 (3.2) | 23.0 (2.9) | 22.1 (3.6) | 0.319 | 0.292 |
| Smoking history | 25 (52.1) | 35 (72.9) | 16 (53.3) | 22 (73.3) | 0.441 | 0.423 |
| Performance status ≥2 | 7 (14.6) | 4 (8.3) | 2 (6.7) | 4 (13.3) | 0.197 | 0.210 |
| High grade | 43 (93.5) | 44 (91.7) | 26 (89.7) | 28 (93.3) | 0.069 | 0.140 |
| Grade missing | 2 (4.2) | 0 (0.0) | 1 (3.3) | 0 (0.0) | 0.295 | 0.236 |
| Variant histology at baseline | 11 (22.9) | 10 (20.8) | 8 (26.7) | 6 (20.0) | 0.050 | 0.161 |
| Values are mean (SD) or n (%). Low/high refers to the median-defined p-LATS1/2 groups. High-grade percentages exclude missing values; missing-grade percentages use the full group denominators. Absolute SMDs use pre-matching denominators. Matching retained 30 pairs, but the prespecified balance criterion (\|SMD\| <0.10 for all five propensity-score covariates) was not met; matched survival analyses were therefore not performed.  BMI, body mass index; NAC, neoadjuvant chemotherapy; p-LATS1/2, phosphorylated large tumor suppressor kinases 1 and 2; SD, standard deviation; SMD, standardized mean difference. | | | | | | |

Table S2 Exploratory 36 month overall survival ROC analysis

| **Measure** | **Estimate** |
| --- | --- |
| **Cohort and continuous marker discrimination** | |
| Patients | 96 |
| Deaths over all follow-up | 43 |
| Deaths by 36 months | 33 |
| Observed survival beyond 36 months | 43 |
| Censored at or before 36 months | 20 |
| Continuous-marker AUC | 0.615 |
| Bootstrap 95% CI for AUC | 0.485–0.739 |
| **Selected cutoff performance** | |
| Sensitivity (apparent) | 0.593 |
| Sensitivity (mean optimism) | 0.051 |
| Sensitivity (optimism corrected) | 0.541 |
| Specificity (apparent) | 0.628 |
| Specificity (mean optimism) | 0.034 |
| Specificity (optimism corrected) | 0.594 |
| Youden (apparent) | 0.220 |
| Youden (mean optimism) | 0.085 |
| Youden (optimism corrected) | 0.135 |
| **Bootstrap cutoff stability** | |
| Successful resamples | 2000 / 2000 |
| Selected cutoff median | 45.006 |
| Selected cutoff interquartile range | 28.297–187.664 |
| Selected cutoff 2.5th–97.5th percentiles | 7.125–202.279 |
| Distinct selected cutoff values | 87 |
| Exact original cutoff selected again | 16.55% |
| Original 38/58 split selected again | 30.65% |
| Selected cutoff above original median | 30.50% |
| Time-dependent ROC analysis evaluated all-cause mortality at 36 months after RC using inverse-probability-of-censoring weighting. The exploratory cutoff was 45.006, with lower scores indicating higher risk (≤45.006: n = 38; >45.006: n = 58). Cutoff selection maximized the Youden index with at least 20 patients per group and was repeated in 2,000 bootstrap resamples. The AUC CI is a percentile bootstrap interval; cutoff percentile ranges describe selection variability, not confidence intervals for a clinical threshold. Optimism-corrected estimates reflect internal validation only. The original median-defined groups were retained in the main analyses.  AUC, area under the curve; CI, confidence interval; p-LATS1/2, phosphorylated large tumor suppressor kinases 1 and 2; RC, radical cystectomy; ROC, receiver operating characteristic. | |

Table S3 Model diagnostics and sensitivity summaries

| **Analysis** | **Model or p-LATS1/2 form** | **Statistic** | **OS** | **PFS** |
| --- | --- | --- | --- | --- |
| Proportional hazards | Base, n = 96 Continuous | Global P | 0.2733 | 0.1272 |
|  |  | p/ypT P | 0.0297 | 0.0652 |
|  |  | p-LATS1/2 P | 0.2276 | 0.1191 |
| Proportional hazards | Base, n = 95 Continuous | Global P | 0.2695 | 0.1102 |
|  |  | p/ypT P | 0.0263 | 0.0600 |
|  |  | p-LATS1/2 P | 0.2264 | 0.1223 |
| Proportional hazards | Extended, n = 95 Continuous | Global P | 0.4281 | 0.2512 |
|  |  | p/ypT P | 0.0698 | 0.2109 |
|  |  | p-LATS1/2 P | 0.2645 | 0.1607 |
| Proportional hazards | Base, n = 96 Binary | Global P | 0.2694 | 0.1360 |
|  |  | p/ypT P | 0.0222 | 0.0526 |
|  |  | p-LATS1/2 P | 0.2653 | 0.1789 |
| Proportional hazards | Base, n = 95 Binary | Global P | 0.2677 | 0.1200 |
|  |  | p/ypT P | 0.0190 | 0.0472 |
|  |  | p-LATS1/2 P | 0.2800 | 0.1916 |
| Proportional hazards | Extended, n = 95 Binary | Global P | 0.4882 | 0.3438 |
|  |  | p/ypT P | 0.0502 | 0.1765 |
|  |  | p-LATS1/2 P | 0.3809 | 0.3670 |
| Proportional hazards | Interaction, n = 96 Continuous | Global P | 0.4438 | — |
|  |  | p/ypT P | 0.0321 | — |
|  |  | p-LATS1/2 P | 0.2395 | — |
| Proportional hazards | Interaction, n = 96 Binary | Global P | 0.3871 | — |
|  |  | p/ypT P | 0.0244 | — |
|  |  | p-LATS1/2 P | 0.3071 | — |
| Nonlinearity | Extended Continuous | Global P | 0.910 | 0.964 |
|  |  | Age P | 0.926 | 0.878 |
|  |  | p-LATS1/2 P | 0.742 | 0.727 |
|  |  | Ki-67 P | 0.475 | 0.641 |
| Model information | Base | Patients | 96 or 95 | 96 or 95 |
|  |  | Events | 43 | 47 |
|  |  | Coefficients | 5 | 5 |
|  |  | Events per coefficient | 8.60 | 9.40 |
| Model information | Extended | Patients | 95 | 95 |
|  |  | Events | 43 | 47 |
|  |  | Coefficients | 7 | 7 |
|  |  | Events per coefficient | 6.14 | 6.71 |
| Leave one out | Extended Continuous | p-LATS1/2 HR range per 1 point | 0.99459–0.99622 | 0.99552–0.99683 |
|  |  | Wald P value range | 0.00747–0.05323 | 0.01373–0.07233 |
| Discordant LVI exclusion | Per 1 H-score point | n / events | 94 / 42 | 94 / 46 |
|  |  | HR (95% CI) | 0.9955  (0.9917–0.9993) | 0.9963  (0.9928–0.9998) |
|  |  | P value | 0.020 | 0.036 |
| Discordant LVI exclusion | Low vs high | n / events | 94 / 42 | 94 / 46 |
|  |  | HR (95% CI) | 1.7658  (0.8648–3.6054) | 1.6842  (0.8561–3.3133) |
|  |  | P value | 0.118 | 0.131 |
| Nested extension | Continuous | Likelihood ratio χ² | 7.378 | 8.858 |
|  |  | df | 2 | 2 |
|  |  | P value | 0.025 | 0.012 |
| Nested extension | Binary | Likelihood ratio χ² | 6.973 | 9.000 |
|  |  | df | 2 | 2 |
|  |  | P value | 0.031 | 0.011 |
| Model specifications are provided in Tables 2–4. Sensitivity-analysis HRs refer to p-LATS1/2. The LVI sensitivity analysis excluded one patient with discordant total and component LVI records. Nested likelihood-ratio tests compared base and extended models in the same 95 patients. —, not performed.  CI, confidence interval; df, degrees of freedom; HR, hazard ratio; LVI, lymphovascular invasion; OS, overall survival; p-LATS1/2, phosphorylated large tumor suppressor kinases 1 and 2; PFS, progression-free survival; p/ypT, pathological T category without/after neoadjuvant therapy. | | | | |
